# Early trauma and psychotic experiences in autism: a neurodevelopmental framework integrating allostatic overload, symbolisation, and stress reactivity

**DOI:** 10.3389/fpsyt.2026.1898132

**Published:** 2026-09-24

**Authors:** Antonella Trotta

**Affiliations:** 1 Institute of Psychiatry, Psychology & Neuroscience, King’s College London, London, United Kingdom; 2 School of Health and Social Care, University of Essex, Colchester, United Kingdom

**Keywords:** aberrant salience, autism spectrum disorder, childhood trauma, meaning-making, neurodevelopment, psychotic experiences, stress reactivity, symbolisation

## Abstract

Adverse childhood experiences are common worldwide and are associated with enduring mental and physical health consequences. Autism, a complex and heterogeneous neurodevelopmental condition shaped by both genetic and environmental influences, has a global prevalence of approximately 1% and is associated with increased exposure to adversity, including bullying, social exclusion, and interpersonal misattunement. Such exposures may partially mediate associations between autistic traits and later psychotic experiences independently of genetic liability. Developmental trauma is increasingly recognized as an environmental factor that may shape the emergence of psychotic experiences in autism by interacting with neurodevelopmental differences. Neurobiological findings in trauma-exposed populations implicate dysregulated stress-response systems and alterations in circuits supporting threat detection and affect regulation, including heightened amygdala reactivity, reduced hippocampal volume linked to impaired contextual processing, and altered ventromedial prefrontal functioning. Integrating epidemiological, neurobiological, and developmental perspectives, this paper proposes a framework linking three interrelated mechanisms: (i) dissociation and reduced integration of affective and relational experience, (ii) constraints on symbolisation and narrative meaning-making, and (iii) heightened stress reactivity that amplifies threat-based processing and alters salience attribution. In autistic individuals, pre-existing differences in sensory processing and intersubjective functioning may compound the impact of adversity, lowering the threshold at which anomalous experiences acquire distressing or aberrant meaning, particularly during sensitive developmental periods such as childhood and adolescence. This article is intended as a Perspective that brings together findings from autism, trauma, psychosis, and developmental psychology to generate a theoretical framework and set of testable hypotheses. It does not present a systematic review of the literature or seek to establish causal relationships but rather proposes potential mechanisms through which early trauma may influence the emergence of psychotic experiences in a subgroup of autistic individuals. The model presented highlights trauma as a modifiable environmental pathway with implications for trauma-informed assessment, early intervention, and future multilevel research.

## 1 The dialectic between nature and nurture in autism

Autism spectrum disorder (ASD) affects approximately 1 in 127 individuals globally, although prevalence estimates vary across regions and methodologies, reflecting differences in diagnostic practices, service provision, and socio-environmental contexts (1). ASD is a multifaceted neurodevelopmental condition characterised by early-emerging differences in social communication and restricted or repetitive patterns of behavior (2). From early in development, reduced attention to social stimuli, including faces, voices, and gestures, has been observed in infants who later receive a diagnosis of autism, with subsequent implications for social development (3–5). Advances in autism research have led to a growing recognition of autism as a lifelong and heterogeneous form of neurodivergence, characterised by substantial individual variation, frequent underdiagnosis in females, and high rates of co-occurring mental health conditions (6).

Contemporary research also indicates that ASD has a substantial but highly heterogeneous genetic basis, involving the combined contribution of numerous genetic variants and polygenic influences (7–10). These genetic factors converge on pathways related to neurodevelopment, gene expression regulation, and neuronal communication, yet the mechanisms through which genetic liability translates into cognitive and behavioral outcomes remain only partially understood (11). Increasingly, genetic influences are conceptualised within broader models of epigenetic modulation and gene–environment interaction, suggesting that genetic liability confers vulnerability rather than determining fixed developmental outcomes (10).

Exposure to adversity in childhood is common, with approximately two-thirds of children experiencing at least one significant adverse event (12). Evidence indicates that children diagnosed with autism are more exposed to adversity, being more than twice as likely as children without autism to experience adverse childhood experiences (ACEs; 13), particularly bullying, peer victimisation, and family-related stressors (14–18). Kerns et al. (19) similarly argue that children with autism may be especially vulnerable to potentially traumatic experiences because of differences in sensory processing, social understanding, and regulation. These observations make it important to consider early adversity as a key environmental factor that may interact with neurodevelopmental differences to shape later psychopathological trajectories (20). A central question is not only whether children with autism are more likely to encounter adversity, but *how* adversity is likely to be experienced and incorporated into development. This question is especially important when considering psychotic experiences, because such experiences often emerge at the point where perception, affect, interpersonal meaning, and stress responsivity become difficult to integrate.

In this paper, I distinguish between adversity as a broad category of harmful developmental experience, ACEs as operationalised epidemiological exposures, and trauma as experience that is subjectively overwhelming and difficult to integrate. The terms developmental trauma and interpersonal trauma are used more specifically to describe chronic or repeated relational adversity during childhood that may affect attachment, regulation, and the organisation of self-experience (21). Psychotic experiences refer to hallucination-like, perceptual, or delusion-like experiences involving altered interpretations of reality (22, 23). They are distinguished from trauma-related intrusions, dissociative states, sensory processing differences, and social misunderstandings, although these phenomena may interact and contribute to vulnerability under conditions of stress or adversity.

## 2 Conceptual model

Early trauma may interact with autism-related neurodevelopmental differences through two closely related pathways: stress-system dysregulation and impaired integration of experience. Within this framework, genetic and developmental factors influence domains such as sensory processing, intersubjective integration, and symbolic functioning, while adverse experiences act as environmental stressors that may amplify these differences across development. The framework presented here should be understood as hypothesis-generating rather than evidentiary. Although it draws on findings from epidemiological, developmental, neurobiological, and clinical research, the purpose of this Perspective is not to provide a systematic synthesis of evidence. Instead, it seeks to integrate observations from multiple disciplines into a coherent conceptual model that can inform future empirical investigation.

Developmental trauma is generally understood as exposure to multiple, chronic, and developmentally adverse interpersonal experiences during childhood, including abuse, neglect, bullying, and caregiving disruption, that interfere with normative emotional, cognitive, relational, and neurobiological development (21, 24). Chronic trauma may interfere with neurobiological development and compromise the integration of sensory, emotional, and cognitive experience, thereby contributing to fragmented or poorly organized mental states (25, 26). Repeated exposure to overwhelming or poorly integrated experience may dysregulate stress-response systems and contribute to allostatic overload (27), with cascading effects on affect regulation, threat processing, and perceptual salience (28–30). In parallel, trauma may constrain the capacity for symbolisation and foster dissociative modes of functioning. When symbolisation is compromised, experience is more likely to remain concrete, fragmented, or bodily based rather than becoming integrated into meaningful psychological form (31, 32).

A substantial body of research suggests that childhood abuse, neglect, and failures of care in childhood may contribute to dissociation, limiting the individual’s capacity to integrate thoughts, feelings, memories, and perceptions into a cohesive sense of self (33, 34). Allen (35) describes two core dimensions of dissociation: detachment from the overwhelming experience and compartmentalization. Detachment could be described as an altered state of consciousness characterized by a sense of separation from aspects of ordinary experience’ (36), including feeling disconnected, unreal, or as if in a dream (37, 38). Compartmentalisation refers to a lack of integration between mental processes, such as emotions, memory, cognition, and identity, that are normally linked (39).

Although dissociation may function in the short term as an adaptive response to threat it may become debilitating if it persists over time and contributes to enduring anxiety, fragmentation, or difficulties with self-organisation (33, 40, 41). Developmental trauma may give rise to mental states that remain insufficiently integrated into the person’s broader sense of self, even when the individual does not meet criteria for Developmental Trauma Disorder or a dissociative disorder (42). From the perspective of structural dissociation, aspects of psychological functioning may become organized with a degree of autonomy from other parts of the self, particularly in response to trauma-related cues or relational contexts (43). These states tend to be activated in specific situations that resonate with earlier traumatic experience. When activated, they may bring together traumatic memories, affective states, defensive behaviors, bodily arousal, and relational expectations into a relatively self-contained mode of functioning (42, 44, 45).

These interacting processes may lower the threshold at which anomalous experiences acquire aberrant salience (46, 47), thereby increasing vulnerability to psychotic experiences in a subgroup of autistic individuals.

This Perspective therefore adopts a transdiagnostic framework, in which trauma functions as a potentially modifiable pathway through which autism-related neurodevelopmental differences may be intensified, organized, or redirected over time. Throughout this paper, autism is understood as a heterogeneous neurodevelopmental condition rather than as a pathological state; the proposed model concerns how early adversity may interact with autism-related differences in a subgroup of individuals, not autism as a causal pathway to psychosis.

Furthermore, the mechanisms linking trauma and psychotic experiences might not be unique to autism. Stress reactivity, allostatic overload, altered threat processing, predictive processing disturbances, and aberrant salience attribution have all been described in broader models of trauma and psychosis. The autism-specific contribution of the present framework concerns how autism-related differences in sensory processing, contextual integration, social communication, interpersonal understanding, and symbolic meaning-making may influence the appraisal, regulation, and integration of adverse experiences. For example, heightened sensory sensitivity, difficulties contextualising social experiences, reduced access to interpersonal co-regulation, and a more detail-focused processing style may increase the likelihood that distressing experiences remain fragmented, concrete, or difficult to incorporate into coherent autobiographical narratives. The present model therefore proposes autism-specific modifiers of general trauma–psychosis pathways rather than a distinct autism-specific mechanism of psychosis. Under conditions of adversity, these developmental differences may shape how threatening experiences are encoded, interpreted, and integrated, thereby influencing vulnerability to later psychotic experiences in a subgroup of autistic individuals.

## 3 Why trauma may be experienced differently in autism

Trauma cannot be understood solely in terms of exposure. Contemporary relational and developmental models conceptualise trauma as a disruption in the capacity to integrate experience into coherent representations of self and others. From this conceptualisation, the impact of trauma depends not only on what happens, but also on how an experience is appraised, regulated, and symbolically processed (32, 48).

This distinction is particularly important in autism. Differences in perceptual and information processing, including reduced sensitivity to social context or global meaning, may alter how events are appraised, increasing the likelihood that they are experienced as confusing, overwhelming, or traumatic (49). Such differences may also contribute to a reduced sense of agency and reliance on less adaptive coping strategies, including repetitive behaviors, in place of cognitive reappraisal or problem-solving (50, 51). In this way, autism may shape not only exposure to adversity but also the way adversity is interpreted, regulated, and integrated over time. Clinical and developmental observations further suggest that similar events may have markedly different psychological consequences depending on how they are processed within the context of neurodevelopmental differences (19).

Developmental findings support this view. Infants who later develop autism, as well as those within the broader autism phenotype, show subtle divergences in their interactions with caregivers during the latter part of the first year of life (52, 53). The most consistent findings concern delays in preverbal communication, such as reduced use of gestures, limited prelinguistic vocalisations, and impaired coordination of vocal and gestural signals, and lower levels of interactive reciprocity or mutual engagement (54–56). These early differences may reflect emerging constraints in intersubjective integration, which are central to later difficulties in symbolisation and meaning-making, the process by which sensory, affective, cognitive, and relational experiences are organized into coherent representations and narratives that help individuals understand themselves, others, and their environment. Importantly, these adjustments do not in themselves constitute trauma. However, they may shape the developmental conditions under which safety, attunement, and regulation are experienced. In this sense, early differences in reciprocity and communication may create a context in which later adversity is more difficult to process and integrate. A child who perceives relational experiences as less predictable, less comprehensible, or less regulating may be more vulnerable to registering stressful events as overwhelming and less able to rely on interpersonal contexts for repair (19). Attachment research further suggest that trauma unfolds within interpersonal contexts (57–59). Internal experience may come to be organized into multiple self-states associated with distinct affective configurations and relational expectations, reflecting early patterns of self–other interaction (60). Under conditions of threat, dissociative processes may limit the integration of these states, while trauma may be experienced as disconnection, distrust, and a loss of safety in relation to others (61). In autism, where reciprocity and intersubjective attunement may already be more difficult to establish, such relational disruptions may be especially consequential.

In neurodevelopmental terms, symbolic functioning can be understood as the capacity to convert sensory, affective, and relational experience into mental representations that can be reflected on, communicated, and integrated into thought. Segal (62) distinguished symbolic thinking from what she defined symbolic equation in which the symbol is treated as if it were identical to the object or experience it refers to. For example, after a distressing interpersonal event, a person may be able to think about it, describe it, and link it to feelings of fear or loss. However, when symbolic functioning is compromised, the experience may instead remain concrete and immediate, so that an ambiguous interaction is felt not as a reminder of danger but as danger itself. She proposed that symbolic function develops as separateness and absence become more tolerable, making it possible to use representations flexibly rather than responding to them in a concrete, immediate way. When this developmental achievement is compromised, as may occur in psychotic states, symbols become less available for communication and reflective thinking because they are apprehended as concrete equivalents rather than representations. Segal further links symbol formation to the capacity for communication, both with others and within the mind itself, suggesting that disturbances in symbol formation compromise not only expression but also thinking.

This implies that symbolic function is not merely a cognitive achievement but a relational and affective one: it emerges through the capacity to survive separateness without catastrophic loss of coherence. Within the present framework, early trauma may interfere with this developmental achievement by intensifying threat, disrupting integration, and increasing the likelihood that experience remains concrete, sensory-bound, or dissociated rather than symbolically elaborated.

## 4 From trauma to psychotic experiences in autism: a multilevel mechanism

Individuals with a diagnosis of autism are at increased risk of psychotic experiences and psychotic disorders relative to the general population (63). Meta-analytic work suggests that people with autism are over three times more likely to have schizophrenia than controls (64), while pooled estimates indicate elevated rates of psychosis and psychotic experiences in autistic adults (65). Increased paranoid thoughts have also been reported (66). Epidemiological evidence further suggests that childhood trauma partially mediates the association between autistic traits and later psychotic experiences, independently of genetic liability (67). Taken together, these findings indicate that environmental adversity may play a significant role in shaping how neurodevelopmental differences are expressed over time.

### 4.1 Dissociation and failure of integration

Relational and developmental formulations increasingly conceptualise trauma not simply as exposure to threat, but as a disruption in the capacity to integrate experience into coherent representations of self and world. Following Cardeña et al. (68), dissociation can be understood as a broad construct encompassing: (i) compartmentalisation, in which mental contents or systems are not integrated into conscious awareness; (ii) detachment, involving disconnection from the self or environment; and (iii) a defensive process through which overwhelming experience is kept apart from ordinary mental integration. This distinction is important because dissociation is not equivalent to psychosis, but may contribute to psychotic vulnerability when unintegrated experience becomes difficult to symbolise, contextualise, or communicate.

From this perspective, trauma is closely linked to dissociation, understood as a failure of integration that restricts access to aspects of experience and limits the capacity to hold affective, perceptual, and relational experience together in a meaningful way (32). This formulation has particular relevance in autism, where symbolic and intersubjective integration may already be developmentally constrained. Trauma may intensify existing difficulties in integrating affective, perceptual, and relational experience, increasing reliance on concrete or fragmented modes of processing (69). What is lost is not only narrative coherence, but the capacity to place experience within a broader interpersonal and symbolic field. In this sense, dissociation may be understood not simply as a symptom or defense, but as a developmental compromise in the face of overwhelming experience.

### 4.2 Allostatic overload and stress reactivity

Singletary’s formulation provides an important extension of this account by conceptualising ASD as shaped, in part, by early environmental deprivation, chronic stress, and cumulative allostatic overload (30). Neurobiological vulnerabilities may predispose the child to experience sensory and interpersonal input as aversive, resulting in heightened arousal and disrupted social engagement. This may initiate a self-reinforcing cycle in which reduced interaction limits opportunities for social learning and diminishes access to regulatory caregiving. Even in the context of sensitive caregiving, social experience may be misinterpreted as threatening or unrewarding, contributing to a relative deprivation of social–emotional input.

Over time, this pattern may lead to sustained overarousal, heightened stress reactivity, and maladaptive coping, culminating in allostatic overload. In this framework, early deprivation and traumatic stress are not simply external events but processes that shape the organisation of experience, influencing both the observable features of ASD and the individual’s subjective world. The child’s difficulty is not only that stress occurs, but that the means of using relationships, symbolisation, and reflective thought to regulate stress are themselves compromised.

From this perspective, allostatic overload may be conceptualised as one candidate pathway through which repeated adversity could influence stress sensitivity and threat processing, thereby contributing indirectly to vulnerability for psychotic experiences.

### 4.3 Symbolisation, salience, and psychotic meaning-making

Heightened stress reactivity may amplify threat processing and contribute to aberrant salience attribution, while failures of integration and reduced symbolisation may limit the capacity to metabolise intense affective and perceptual experience. As a result, anomalous experiences may be perceived as intrusive, concrete, or externally generated.

Frances Tustin’s formulation offers a particularly useful bridge between early relational trauma and psychotic phenomena in autism. She describes the infant’s premature experience of separateness from the caregiver as a catastrophic rupture in bodily continuity rather than a manageable developmental transition (70–72). In this framework, separateness may be registered as an intrusive impingement that overwhelms the infant’s capacity for integration and generates profound anxiety. Delusional systems may then emerge as defensive organisations designed to restore continuity and coherence in the face of unbearable rupture. This formulation resonates closely with contemporary accounts of trauma as a failure of integration and with models of psychosis that emphasise stress sensitivity and aberrant salience processing as mechanisms through which vulnerability becomes symptom expression. Read in this way, Tustin’s account helps connect early bodily and relational experience with later disturbances in meaning-making. If separateness is encountered not as a tolerable differentiation but as catastrophic rupture, then defensive attempts to preserve coherence may take forms that are concrete, sensory-based, or delusional. This makes her work especially relevant to a model in which psychotic experiences emerge when overwhelming experience cannot be symbolised and instead becomes organized around threat, intrusion, and externalisation.

The concept of aberrant salience is drawn from psychosis research, where dysregulated dopaminergic signaling has been proposed to lead neutral or ambiguous stimuli to be experienced as unusually significant or personally meaningful. In the present paper, this concept is used to describe a possible final common pathway through which stress reactivity and impaired integration may influence psychotic meaning-making; it is not assumed that this pathway has been directly demonstrated in autistic individuals exposed to trauma. This account is also compatible with predictive processing models of psychosis, in which chronic uncertainty and heightened threat sensitivity may bias the interpretation of ambiguous stimuli towards personally significant or threatening meanings (73).

In individuals with a diagnosis of autism who are exposed to trauma, altered appraisal and impaired integration may therefore interact with stress reactivity to increase the likelihood that anomalous experiences acquire aberrant salience. Psychotic experiences in autism can thus be understood as one possible outcome of interacting vulnerabilities in integration, symbolisation, and stress responsivity.

## 5 Neurobiological correlates

Contemporary neuroimaging findings have challenged earlier assumptions that autism is characterised by clear-cut or qualitatively distinct patterns of brain structure. Large-scale MRI studies and meta-analyses involving hundreds of autistic participants have revealed relatively little evidence for neuroanatomical features that clearly distinguish autistic from non-autistic individuals at a categorical level. Instead, the emerging picture is one of subtle and distributed quantitative variation, with differences in the size, connectivity, or development of multiple brain regions rather than discrete anatomical abnormalities (6, 49).

This shift from categorical to dimensional accounts of autism has important implications for understanding trauma processing. Consistent with the autism-specific modifiers outlined above, neuroimaging findings suggest that autism may be better understood in terms of differences in information processing rather than discrete anatomical abnormalities.

Happé and Frith’s weak central coherence account proposes that autistic individuals often show a bias toward local rather than global processing, with a tendency to attend to constituent details without automatically integrating them into a broader contextual framework. Importantly, this is increasingly understood as a cognitive style or processing bias rather than a deficit per se (6). For example, childhood trauma has been associated with heightened amygdala reactivity, reduced hippocampal volume, and altered prefrontal functioning, reflecting increased threat sensitivity and impaired regulatory capacity (26, 29).

Such differences may be relevant to how adversity is experienced and integrated. Traumatic events do not consist solely of objective occurrences but also require the individual to contextualise sensory, emotional, interpersonal, and autobiographical information into a coherent representation of what has happened. If experience is processed in a more detail-focused and less contextually integrated manner, overwhelming events may be more difficult to organize into coherent narratives or relational meanings. This does not imply that autism itself causes trauma-related difficulties. Rather, it suggests that autism-related differences in perceptual and cognitive processing may shape the manner in which adverse experiences are appraised, interpreted, and integrated, potentially influencing vulnerability to later distress under certain environmental conditions (74).

Neurobiological research supports this multilevel account by linking early adversity to alterations in systems involved in threat detection and affect regulation. These findings provide a plausible biological pathway through which early adversity may shape the development and persistence of psychotic experiences in neurodevelopmentally vulnerable individuals. Such evidence is consistent with the present model, in which trauma-related stress dysregulation and impaired integration jointly influence perception, affect, and meaning-making. Heightened threat responsivity, reduced contextualisation, and altered salience attribution may together contribute to the emergence of psychotic phenomena under conditions of developmental vulnerability. Neurobiology does not replace psychological explanation here; rather, it supports a multilevel account in which biological, affective, and symbolic processes are developmentally intertwined.

These findings might tentatively suggest biologically plausible mechanisms through which early adversity may influence threat sensitivity, contextual processing, and affect regulation in developmentally vulnerable individuals. Such neurobiological findings should therefore be treated as convergent but indirect evidence, supporting the plausibility of the model while highlighting the need for longitudinal and autism-specific mechanistic research.

## 6 Research and clinical implications

This framework underscores the importance of trauma-informed care in autism, with assessment and intervention approaches that recognize both the high likelihood of adverse experiences and their effects on stress regulation, meaning-making, and relational functioning. It also highlights the relevance of early intervention, particularly in children with autism and adolescents showing signs of trauma exposure, emotional dysregulation, or unusual perceptual experiences (75). Timely intervention may reduce downstream risk for persistent distress, maladaptive coping, and psychotic phenomena by targeting modifiable pathways of stress dysregulation and impaired integration (76).

More broadly, this model suggests that clinicians should attend not only to exposure history, but also to how traumatic experience has been appraised, embodied, and woven into the individual’s developing sense of self and others. In practice, this means that trauma-informed work in autism may need to address sensory and relational processing, not simply traumatic content in narrative form.

Several limitations follow from the integrative nature of this Perspective. First, the proposed links between biological stress systems, integration, symbolisation, and psychotic experiences are not yet supported by a unified empirical literature. Second, much of the neurobiological evidence derives from studies of childhood adversity or psychosis more broadly rather than autistic individuals with trauma exposure and psychotic experiences specifically. Third, concepts such as symbolisation, meaning-making, and integration require further operationalisation and empirical testing.

Future research would benefit from collaboration with autistic individuals at all stages of the research process to ensure that concepts such as trauma and psychotic experiences reflect lived experience as well as clinical theory. Participatory research approach (77) involving autistic individuals in the design, conduct, interpretation, and dissemination of research may improve the validity, acceptability, and translational value of future studies.

## 7 Conclusions

This paper has proposed a multilevel neurodevelopmental model in which early trauma may interact with autism-related neurodevelopmental differences to increase the likelihood of psychotic experiences in a subgroup of autistic individuals. In this framework, genetic and developmental factors shape vulnerability in domains such as sensory processing, intersubjective integration, and symbolic functioning, while adverse experiences act as environmental influences that amplify this difference over time.

Repeated adversity may increase stress sensitivity and affect the organisation of experience across development. In parallel, trauma may constrain symbolisation and foster dissociative modes of functioning, reducing the individual’s ability to integrate affective, perceptual, and relational experience into coherent meaning. These interacting processes may lower the threshold at which anomalous experiences acquire aberrant salience and are interpreted in concrete, intrusive, or externally generated ways.

This framework does not imply a deterministic progression from autism to psychosis, but rather, it highlights early trauma as a modifiable environmental pathway through which neurodevelopmental vulnerability may be shaped towards or away from psychotic outcomes (58). That, in turn, has implications not only for theory, but also for prevention, early identification, and trauma-informed clinical care. Future longitudinal, mechanistic, and experimental research will be required to evaluate the specific pathways proposed and to determine the extent to which the concepts of integration, symbolisation, stress reactivity, and salience attribution contribute to psychosis risk in autistic populations.

## Data Availability

The original contributions presented in the study are included in the article/supplementary material. Further inquiries can be directed to the corresponding author.
